# Social networking in nursing education: integrative literature review

**DOI:** 10.1590/1518-8345.1055.2709

**Published:** 2016-07-04

**Authors:** Luciana Emi Kakushi, Yolanda Dora Martinez Évora

**Affiliations:** 1 Doctoral Student, Escola de Enfermagem de Ribeirão Preto, Universidade de São Paulo, PAHO/WHO Collaborating Centre for Nursing Research Development, Ribeirão Preto, SP, Brazil.; 2 PhD, Full Professor, Escola de Enfermagem de Ribeirão Preto, Universidade de São Paulo, PAHO/WHO Collaborating Centre for Nursing Research Development, Ribeirão Preto, SP, Brazil.

**Keywords:** Social Networking, Education, Nursing

## Abstract

**Objective:**

to identify the use of social networking in nursing education.

**Method:**

integrative literature review in the databases: LILACS, IBECS, Cochrane, BDENF, SciELO, CINAHL, Scopus, PubMed, CAPES Periodicals Portal and Web of Science, using the descriptors: social networking and nursing education and the keywords: social networking sites and nursing education, carried out in April 2015.

**Results:**

of the 489 articles found, only 14 met the inclusion and exclusion criteria. Most studies were published after 2013 (57%), originating from the United States and United Kingdom (77.8%). It was observed the use of social networking among nursing students, postgraduate students, mentors and nurses, in undergraduate programmes, hybrid education (blended-learning) and in interprofessional education. The social networking sites used in the teaching and learning process were Facebook (42.8%), Ning (28.5%), Twitter (21.4%) and MySpace (7.1%), by means of audios, videos, quizzes, animations, forums, guidance, support, discussions and research group.

**Conclusion:**

few experiences of the use of social networking in nursing education were found and their contributions show the numerous benefits and difficulties faced, providing resourses for the improvement and revaluation of their use in the teaching and learning process.

## Introduction

Web-based social networking are activities resulting in connections and interactions between individuals and groups using a wide variety of tools. These tools include email, blogs, instant messaging, text messages and posts as well as programs that enable digital information sharing in video, audio or text format. Social networking websites integrate these tools in easy to handle formats and allow users to determine how and with whom they will share their information^1^.

The increase in the use of social networking, evidently, has become a common phenomenon in recent years and generated great impact on the development of forms of interaction and socialization among students^2^. It is observed that students spend much of their free time in online social networking sites, which represents a great opportunity for educators co-opt their use for academic purposes^3^.

In this way, social networking represents an attractive tool for the educational field, because the students are thoroughly familiar with them and willing to establish more fluid communication with the aim to exchange knowledge, information and ideas^4^.

The educational use of social networking is growing among academics as a powerful tools for teaching and learning^5^. In education, the use of social networking enables the publication and information sharing, self-learning, teamwork, feedback and contact with experts^6^; In adittion, its contributions include the interaction, collaboration, active participation, information, resource allocation and support in educational activities^7^.

Teachers have been engaged in using technology to make learning more personalized, interactive and dynamic, making it easier for students to work with audio, video, interactive games, and more recently, with blogs and social networking^3^.

In short, social networking can be a perfect tool for learning, however, despite the increase in their use, only few students use them for school purposes^6^, highlighting the need to better exploit the potential use of the social networking in higher education^8^.

Having knowledge of the possibilities of the use of social networking in teaching and taking into account the panorama of healthcare education, has social networking technology been used in nursing education? That was the guiding question of this study, with the purpose of knowing the possibilities, ways, successes and failures of their use in this area of study.

## Objectives

To identify the use of social networking in nursing education.

## Method

The integrative literature review was the research method used to achieve the objective of this study, since it has as an advantage the possibility of synthesis and analysis of the scientific knowledge already produced about the research theme.

This research method is characterized by presenting an extensive methodological approach with regard to the literature reviews, allowing the inclusion of multiple studies with different research designs for the complete understanding of the phenomenon studied. This method combines both data of the theoretical literature and empirical, and incorporate wide range of purposes: definition of concepts, review of theories and evidences, and analysis of methodological problems on a particular subject^9^.

The six steps followed to elaborate the integrative review were: definition of the research subject, establishment of the inclusion and exclusion criteria for the literature search, definition of the information to be extracted from the studies, evaluation of the studies included, interpretation of results and data synthesis^10^.

The inclusion criteria for this review were the studies published throughout the period allowed by the selected databases, aiming at including the largest possible number of articles, taking into consideration the contemporaneity of the subject studied.

According to the exclusion criteria, it was removed studies addressing the use of social networking with other approaches, such as:

- in higher education of other professional groups, elementary education, high school education and technical education;
- in research using social networking as a data collection method and interviews;
- in the approaches on the use of social networking as a security method in school environments;
- in the approaches on the user's safety in the use of social networking;
- in the posts and comments from students on social networking;
- regarding to the ethical aspects of the use of social networking;
- in education focused on the patient and on community;
- on the use of other technologies such as Wikis (hypertext), video conferencing, virtual environments, mobile devices, blogs (websites in the format of dairy), YouTube, text messaging, Web sites and simulation of virtual reality.

The studies were resulting from journals indexed in the databases: Latin American and Caribbean Health Sciences (LILACS), Spanish Bibliographic Index of the Health Sciences (IBECS), Cochrane, Brazilian Nursing Databases (BDENF) Scientific Electronic Library Online (SciELO), Cumulative Index to Nursing and Allied Health Literature (CINAHL), Scopus, PubMed, CAPES Periodicals Portal and Web of Science.

A query in the Descriptors of Health Science (MeSH) and in the Medical Subject Headings (MeSH) was performed for the selection of articles, and it was identified and used the descriptors: social networking and nursing education.

Aiming at analyzing any publication related to the theme and in order to expand the study sample, the keywords "social networking sites" and "nursing education" were also used as search strategy, with the main question and the inclusion and exclusion criteria previously established as guidelines to maintain the consistency in the search for articles and avoid possible bias.

In April 2015, therefore, a search using the descriptors and a second research using the keywords were carried out in all databases (Table 1).

**Table 1:** Distribution of the number of articles found in each database, according to the descriptors and keywords used. Ribeirão Preto, SP, Brazil, 2015

Data base	Descriptors: social networking in nursing education	Keywords: social networking sites e nursing education	Total of articles found
LILACS	0	0	0
IBECS	0	0	0
Cochrane	0	0	0
BDENF	0	0	0
SciELO	1	0	1
CINAHL	7	30	37
Scopus	79	28	107
PubMed	75	21	96
CAPES Periodicals Portal	87	124	211
Web of Science	23	14	37
Total of articles found	272	217	489

Firstly, it was carried out the reading of the titles and abstracts of the total sample, taking into account the inclusion and exclusion criteria. Thus, of the total of 489 articles found, 97 articles were selected.

In a second moment, of the 97 articles selected, it was performed the removal of 72 duplicate articles, and the sample consisted of 25 articles.

In the third moment, it was carried out the reading in full of those 25 articles, and 11 articles were removed for not meeting the inclusion and exclusion criteria, thus the final sample was composed of 14 articles.

**Figure 1:**
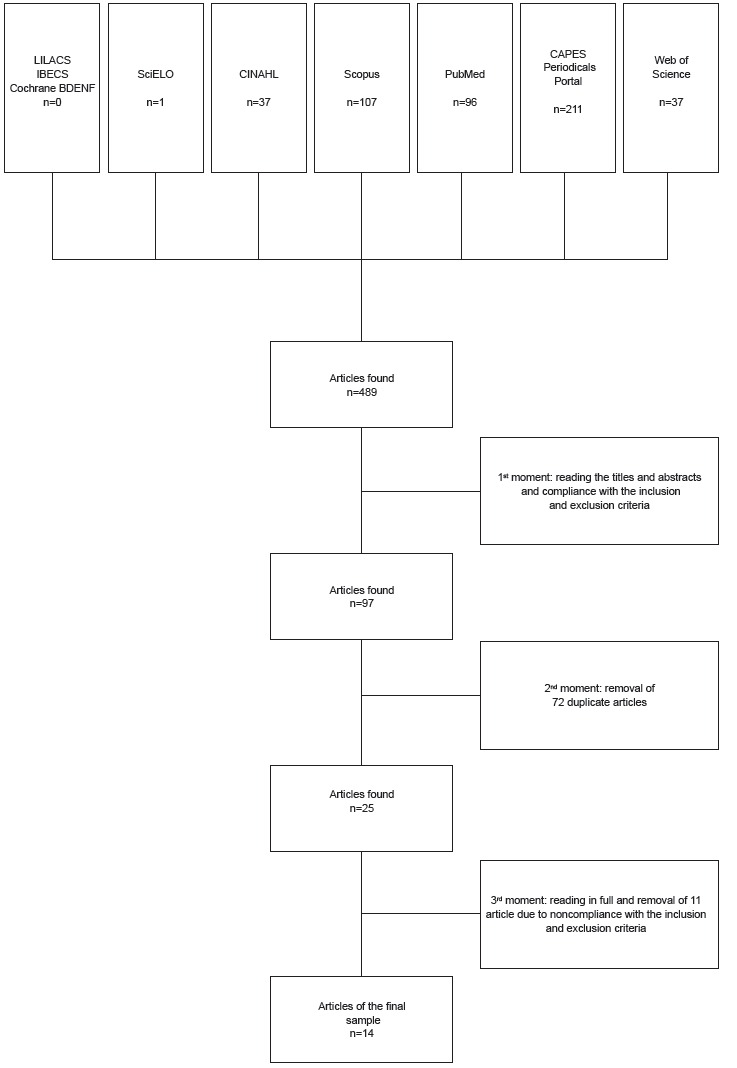
Identification, selection and inclusion of publications in the sample of the integrative review. Ribeirão Preto, SP, Brazil, 2015

Figure 1 below illustrates the process of articles selection of this integrative review.

For the analysis in full of the selected articles, an instrument of collection and synthesis of data was used in order to extract, organize and summarize the information and facilitate the construction of the database.

**Figure 2:**
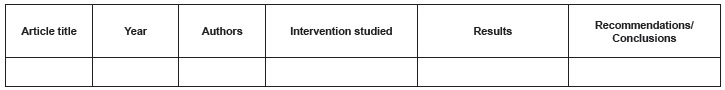
Instrument adapted for collection and synthesis of data^11^. Ribeirão Preto, SP, Brazil, 2015

The instrument developed and validated by Ursi and Galvão^11^, for the collection and analysis of articles in an integrative literature review, was adapted and used in this stage of the study (Figure 2), comprising the following items: article title, year of publication, authors, studied intervention, results and recommendations/conclusions.

**Figure 3:**
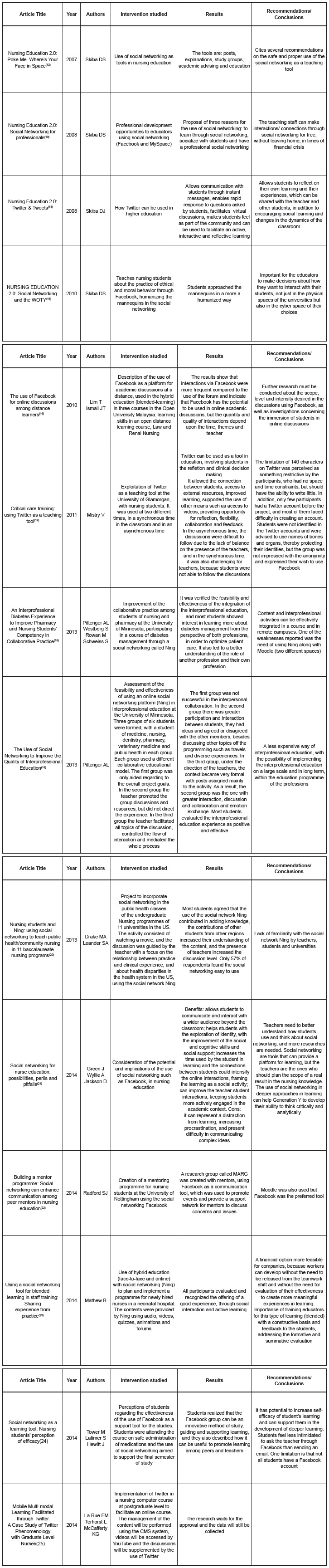
Studies included in the integrative review. Ribeirão Preto, SP, Brazil, 2015

In this integrative review of the 489 articles found, 14 articles met the inclusion and exclusion criteria and composed the sample, as shown in Figure 3.

## Results

In this integrative review, it was found an article in 2007, two in 2008, two in 2010, one in 2011, three in 2013 and five in 2014.

According to the population target of the nursing education, it is emphasized that seven articles cite the use of social networking with nursing students (50%), an article waits for the approval for its use with postgraduate students in nursing (7.1%), an article mentions the use of social networking by nursing mentors (7.1%), an article uses social networking to newly hired nurses in a neonatal hospital (7.1%) and four articles list the benefits of its use in nursing education (28.6%).

Among the seven studies involving nursing students, it was observed that four were performed in undergraduate nursing programmes (57.1%), one was developed in hybrid education involving nursing students (14.3%) and two addressed the interprofessional education among nursing students and other professionals (28.6%).

Regarding the study sites, it was observed that among the articles that used social networking with nursing students, the majority of them (44.5%) were developed in the United States of America (USA), with two studies in Minnesota, one in Arizona and one involving eleven universities in the USA. In addition, there was a study developed in Malaysia, one in Queensland, Australia and another one in Glamorgan, United Kingdom.

The study involved the use of social networking for mentors in nursing and it was developed at the University of Nottingham in UK, and the study involving newly hired nurses of a neonatal hospital was also held in London in UK. In this way, the studies analyzed were developed in several countries and in total, four studies (44.5%) were carried out in the US, three (33.3%) in UK, one was performed (11.1%) in Malaysia and one in Australia.

Analyzing the authors of the studies in this review, it can be observed that almost half of publications on the subject in focus were discussed by two authors: Skiba DS (4: 28.5%) and Pittenger AL (2: 14.2%).

Among the topics covered on the use of social networking with nursing students, it was observed that the topics and subjects were very diversified, and thus it was possible to highlight: information technology and project management, safe administration of medications, disparities in the healthcare system in the US, recommendations for the incorporation of social networking in education, diabetes management, critical care and the practice of ethical and moral behavior.

Social networking sites have been used in different ways for nursing education. Six articles (60%) used them in teaching of a certain content by means of audios, videos, quizzes, animations, forums, guidance and support; three articles (30%) used them only for the discussion after the transmission of the content and 1 (10%) in the formation of a research group.

According to the types of social networking used in the articles included in this review, it is noticed that 6 (42.8%) involved the social network Facebook, 4 (28.5%) used Ning, 3 (21.4%) used Twitter and 1 (7.1%) mentioned the benefits of MySpace.

Regarding the studies that addressed other approaches on the use of social networking with nursing students, it was observed that two described social networking as tools to be used in nursing education, a study reported the benefits of using social networking (Facebook and MySpace) to the educators and two others mentioned the benefits to the students.

The tools used in nursing education were posts, explanations, study groups, academic advising, connection between students, access to external resources, opportunity for reflection, flexibility, collaboration and feedback.

Among the results and benefits cited with the use of Facebook and MySpace, it is highlighted as benefits for the educators the possibility to learn through social networking, interact with students, work in network with other professionals and establish interaction and connection without leaving the house in times of financial crisis.

Regarding the students, the social network Facebook allows a better interaction with and among students, can be used in academic discussions, allows to connect and interact with a wider audience, helps students in the exploration of their professional identity and in the improvement of their social and cognitive skills, allows a social support, increases the time dedicated by the student to learning, can be used in deeper approaches, helping students to develop their ability to think critically and analytically. Additionally, Facebook can also be used in research groups and, finally, it can be considered as a novel method for guiding and supporting learning. Besides the benefits resulting from the use of Facebook, it was also mentioned the difficulties in communicating complex ideas and the possible increasing lack of attention, which can cause procrastination.

With respect to the social network Twitter, it was mentioned that it presents a quick way to communicate with students, facilitates virtual discussion, encourages social learning with changes in the dynamics of the classroom, facilitates active, interactive and reflective learning, enables students to feel as part of the community, allows students to reflect on their own learning and their experiences, sharing them with teachers and other students. However, the use of 140 characters as allowed by this social network was considered as a limitation by the participants, who had no restriction of time or space, however, at this time, they have mentioned it in relation to the number of characters.

Regarding the social network Ning, it was verified visibility and effectiveness in integrating interprofesional education, with the addition of knowledge through the contribution of students of other regions and, consequently, increased understanding of the content addressed. It also allows social interaction and active learning, besides the perception of improvement of the quality of the discussion with the presence of teachers in the social networking. Furthermore, Ning is a less expensive way of interprofessional education, involving various occupational categories separated due to time and space. It should be added the fact that it is feasible for companies in financial terms, with regards to the education of workers, because there is no need to release these workers from the work shift. However, it was not considered easy to handle for almost half of the study participants.

In a study, it was analyzed the form of control by the teachers, on social networking, demonstrating the educator's performance in three different situations. The group without guidance of the teacher failed to achieve the objectives proposed, the group that was completely controlled by the teacher only reached the academic proposal and the group headed by the educator showed more interaction, discussion, collaboration and emotion transmission, when compared to the other groups.

It was also performed the analysis of the teacher's participation at two different times: in a synchronous moment, in which teachers and students were connected in the classroom, and in an asynchronous moment. There were difficulties in both moments. In the synchronous moment, students and teachers were not able to follow the same steps; and in the asynchronous moment, students showed the need of the presence of the teacher.

On the basis of the recommendations of the studies included in this review, it was observed the need for the safe and proper use of social networking as teaching tools, since they are tools that represent a platform for learning, however, teachers are the ones who should plan the scope of an accurate result in the knowledge. Furthermore, educators need to know this kind of learning, addressing the formative and summative evaluation and should decide how to promote the interaction with their students, exceeding the physical space of the universities and reaching the cyber spaces of their choices.

## Discussion

Social networking can provide many opportunities to innovate the teaching and learning process and lead to a reflection on the choice of the methods for assessment of the use of these tools^23^ ^-^ ^24^. However, attention should be paid to the characteristics of each social network and consider the purposes of their use, whether for carrying out discussions, rapid communications, guidance on a job or project, resolution of doubts, or interaction with students, and thus, the educators need to know their features to associate them with their objectives^21^.

It is important to highlight that some studies were conducted in order to provide to students freedom to choose the way of interaction, providing, along with social networking (Facebook, Twitter and Ning), other communication platforms such as Moodle, blogs and forums. Moreover, students gave priority to the access through social network, in relation to the other methods already known in the academic environment^15^ ^-^ ^16^ ^,^ ^18^ ^,^ ^22^.

In addition, it was observed that not all students had, previously, an account in social networking^17^ ^,^ ^24^, which proved to be a limiting factor in the studies, showing the need to consider the familiarity of users with the virtual environment^20^. This familiarity facilitates the user navigation and makes communication so natural that students feel less intimidated to ask any question to the teacher, via social network, compared to sending an e-mail^24^, which demonstrates the presence of a horizontal interaction. Another important aspect is the immersion of students in virtual discussions^16^, highlighting that social networking has the ability to break down the barrier of intimidation, shyness and shame that many students present face to face with the teacher and other colleagues.

It is emphasized the need and importance of the presence of the teacher, both in the synchronous or asynchronous moments, in the use of social networking^17^, since he must realize the needs of students and retake or rearrange strategies in order to assist students in this process. The virtual presence of the teacher is as important as knowing how to manage the form of control of the activities in that environment^19^, allowing freedom of expression of participants in relation to issues beyond the school contents, thus, avoiding to make it a traditional teaching environment.

Other considerations were addressed in the studies analyzed, such as the importance of safety and ethics in the use of social networking^12^. In this sense, a study established the anonymity of the participants, creating imaginary identities, however, the students reported that they would like to have worked with another social network (Facebook)^17^, making clear the importance and the need for people to identify themselves and be identified in a virtual environment.

The types of interaction were used in various ways, with the students, between students, between students and former students, between students of different professions, between students and teachers, between students and experts, between students and professionals, with other audience, showing the possibilty to eliminate the barriers of time and space^12^ ^-^ ^25^. It is also a less expensive and financially viable process of teaching and learning^13^ ^,^ ^19^ ^,^ ^23^, considering the gratuity and freedom of access to social networking.

The benefits of using various social networking in nursing education are immense, both for the students and educators, which includes the interaction, integration and connection among students, socialization with teachers, work in network, discussions, social support, reflection and sharing their own learning and experience, possibility of interprofessional education and achievement of an active, interactive and a reflective learning^13^ ^-^ ^17^ ^,^ ^19^.

Besides allowing interaction with a wider audience, social networking helps students to explore their professional identity, enhancing their social and cognitive skills^21^. It allows a deeper approach, increases the time used for learning, supports the development and social learning^21^ ^,^ ^24^. It can also be used in research groups and as teaching tools, bringing changes in the dynamics of the learning process^12^ ^,^ ^22^.

Social networking is a subject of study that is still beginning to gain space and the analysis of the articles can also identify the difficulties encountered in the use of each social network (Facebook, Twitter and Ning)^17^ ^,^ ^20^ ^-^ ^21^, and its implementation was observed in undergraduate and hybrid (blended) programmes and in interprofessional education, involving students, mentors and nurses^15^ ^-^ ^25^.

The recommendations and gaps of the studies show that more research must still be carried out to better understand the use of social networking by students and understand their immersion in online discussions groups^16^, as well as the safe use of social networking as teaching tools^12^ and the teacher's autonomy in using them^16^ ^,^ ^21^, demonstrating the need to deepen the assessments of their effectiveness for achieving a meaningful learning^23^.

The limitation of this study is related to the small number of international publications found and absence of national publications, which may be related to the keywords used. The social media descriptor, defined in MeSH as a platform that offers the ability and tools to create and publish information accessed through the Internet, was not used in this study because of the lack of articles employing this descriptor in the searches carried out.

## Conclusion

The results of this study show that social network sites used in the process of teaching and learning were Facebook, Ning, Twitter and MySpace, showing the various contributions of their use for the area of education in nursing, such as the benefits to the students, educators, professionals and institutions, besides the tools and the way to use them in the process of teaching and learning.

It was observed that social networking was used to transmit various contents in the nursing field, demonstrating completeness in the transmission and discussion of simple and complex issues, depending on the form of action, control and participation of the teacher. It is also worth mentioning its use in the face-to-face education, distance learning and hybrid education, both the professional and interprofessional learning, proving to be able to be used in many contexts and in many ways, either for the transmission of content, discussions or interaction, depending on the objectives proposed by educators.

The difficulties encountered in the use of each social network, as well as their specificities, contribute to the improvement of the techniques and evaluation of their indication in teaching. Further studies are still required, according to the recommendations and gaps identified in this review, especially in relation to the understanding of the use of social network by students and ways of assessing these tools.
